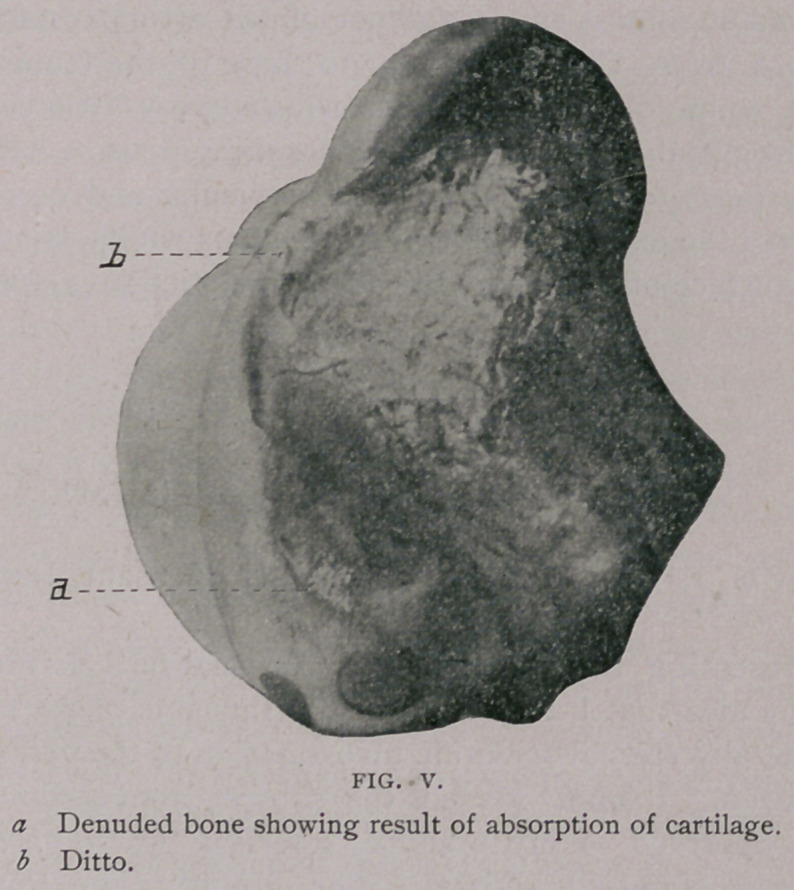# Rheumatism in Horses

**Published:** 1892-12

**Authors:** T. V. Hinebauch


THE JOURNAL
OF
COMPARATIVE MEDICINE AND
VETERINARY ARCHIVES.

Vol. XIII. DECEMBER, 1892. No. 12.

### RHEUMATISM IN HORSES.

By T. V. Hinebauch, M.S., V.S.

Rheumatism in horses we believe to be a neurotic disease, primarily due to improper oxidation or a changed condition of the blood, brought about by improper ventilation, or an over-supply of some of the blood constituents occasioned by improper feed.

During the winter of '91 and '92, a disease passing under the name of "Millet Disease" was prevalent to a considerable extent, and attended by a death rate of 7 to 10 per cent. It received the name "Millet Disease" from the fact that 95 to 98 animals out of every 100 becoming affected had been fed on millet. The symptoms, in the main, were identical with those occurring in animals which did not have access to millet, with the following exception. When millet is fed in considerable quantities it stimulates the kidneys to increased action. The urine is light colored, and the bladder evacuated every two or three hours, large quantities of water being passed at each time. At the time the first symptoms of lameness were noticed the kidneys had almost ceased to act, there being very little urine secreted. When the cause was kept up a sufficient length of time for the reaction to set in, the material which would, under normal conditions, be secreted by the kidneys, is allowed to remain in the system and produce deleterious effects.

It seemed to make no difference as to the condition of the millet with regard to its maturity at the time of cutting. That which was cut when it began to head produced the same effects as that which had already headed out, even if the heads were all matured. During the succeeding winter the disease did not occur to any great extent, there having been but four cases under my immediate care. Three of those had previously been fed on millet, and the fourth was fed on prairie hay, with oats for a grain ration.

*Symptoms*—For several days previous to the attack the kidneys acted very freely. This continued for two or three days, when their action was much less than the normal. Muscles of shoulders, chest, loins and haunches stiff and sore. Later on there is soreness of the joints, usually the stifle and hock. This often changes from one leg to another, or from the hind to the fore extremities. There are well marked symptoms of pain with a slight amount of fever. The temperature usually varies from 102 to 104 degrees. In exceptional instances it may reach 106 degrees, but soon recedes. The pulse is more frequent and hard. The fever is remittant rather than continued, and varies according to the intensity of the pain. At no time is the animal free from pain or fever during the course of the disease. It is a continued succession of ups and downs, gradually growing less marked, until the disease finally comes to an end. The membranes of the eye reddened, tongue coated, mouth hot, dry and sticky, having a peculiar sour odor. Bowels constipated, urine scanty, thick and stringy. The pain in the muscles causes the animal to assume a cramped or drawn together position, with back arched and with a well defined line along the lower ends of the ribs. He has no disposition to move, but if made to do so, has a straddling, ungainly, painful gait, frequently groaning at every step. Occasionally the animal lies down and is unable to rise, more from the severe pain than from any changed condition of the muscles or joints. At first there is profuse sweating, especially in the region of the affected muscles, the animal will flinch and show more or less evidence of pain. Or, if pressure be applied to affected joints, the same result will be manifest. If the horse be down he will lie comparatively quiet and occasionally makes a feeble effort to rise. The intense pain that this movement causes induces quietude again, and it is seldom that he will rise even if persistently urged to do so. There is loss of appetite, the animal generally assuming a painful expression. Should any other region than the one just indicated be affected, similar symptoms will manifest themselves by special movement or manipulation of the diseased part.

When the disease occurs in mares, there is frequently slight tumefaction ot the vulva, which extends into the vagina a variable distance. Underneath the external membrane the connective tissue is engorged with serum, until in some instances the swelling assumes immense volume. Fomenting the swollen parts for a considerable length of time (from one-half to two hours) reduces them to nearly their normal condition. The effect, however, is not permanent, the parts becoming infiltrated in the course of two or three hours. The above condition exists regardless of pregnancy, animals not in foal showing the same condition as those which are in foal. I am at a loss to state what should induce the above symptoms unless it be merely external manifestation of general diseased condition of the generative and urinary organs. Pregnant mares showing the above symptoms do not always abort, but give birth to a healthy foal at the end of the usual period of gestation.

A number of rheumatic cases which came under my observation showed well marked pleuritis and Pleurodenia, the pleurisy being

FIG. I.

*a* Protrusion of Os calcis.

*b* Fiber of Gastrocnemious externus.

very severe and accompanied by a fever raging from 103 to 104 degrees. With vigorous treatment it usuallay subsided within twenty-four to forty-eight hours, the joints then gradually becoming affected until the typical form became established.

DURATION OF ACUTE RHEUMATISM.

There is no definite limit to the time a horse may suffer from a single attack. Some cases recover in five or six days, while others continue for from three to six weeks. Should the acute form terminate in the chronic, it may take several months for the disease to entirely disappear. During that time it will vary in intensity, the pain increasing during cold, damp, and modifying during mild weather. In my experience the disease lasts longer in horses somewhat along in years than in colts. The latter I have found practically well on the fourth day after the attack, and have remained free from the disease up to the present time. It is probable, however, that when the disease continues for a longer time than three or four weeks, it passes into sub-acute, then the chronic form. The duration of acute rheumatism in the horse is more prolonged than in the human subject under the same form of medicinal treatment. This, undoubtedly, is attributable to the surroundings. Though these may be excellent in some instances, for stock, yet they fall far short of what the human family would like to be placed in while suffering from an attack of rheumatism.

After a time, varying from a few days to one or two weeks, the lameness or soreness locates itself permanently. In the majority of cases this is in the posterior half of the body. The muscles of the gluteal region and of the thigh suffering principally when it is of a muscular nature. If the joints become the seat of the disease, the stifle and hock are the ones principally affected.

In describing the various cases to which we shall allude, the history, surroundings and treatment of the animals prior to the attack, symptoms, treatment and termination of the disease will be considered in each individual instance. We will not attempt to describe all cases we have treated, but select several of the first and the last one, as we consider them a fair average of the total number, which, up to the present time, have reached the large number of 168 in fourteen months.

### CASE I.

Bay mare, eight years old, weighs about 1150 to 1200 pounds. In foal, due in two months. Was found lying on her right side. Health previously good. Had never been sick while in possession of present owners, who purchased horse at five years old. Bred and raised in Iowa.

*Surroundings*—Barn low, housed forty head of mules and horses. No special means of ventilation. Ceiling covered with frost, which has been gradually melting and dropping down upon the stock, all of which was more or less wet. Fed on millet in good condition, cut when about one-fifth headed out. Grain consists of bran principally, some receiving one feed of oats per day. During the day the stock ran in a yard, having access to wheat straw.

*Symptoms*—Animal was found lying on her right side, sitting on haunches like a dog, and groaning as though in intense pain, breathing distressed, and number of respirations increased to eighteen per minute, flank and shoulder covered with sweat. Manipulation of hock and stifle joints induced severe pain. Pulse, eighty, wiry and full. Temperature, 104 degrees F. The animal was raised by means of slings, and after remaining in them for half an hour, was able to stand without assistance, but would not

FIG. II.

*I* Depression on articular surface of Patella.

move. The lips of the vulva were much swollen, having a semi-transparent appearance. The evacuation of the bowels showed the secretion to be normal. Urine clear and light colored. The breath had a disagreeable, sour odor.

The case was diagnosed as rheumatism, and the following treatment prescribed: Fomentation of the vulva until swelling disappears. Pure water containing nitrate of potash in quantity sufficient to allow two ounces per day. Hay and bran, dry bedding. Use of slings, and internally:

R. Sodium Salicylate, 2 oz.
Aqua, 8 oz. M.
Sig.: Give one ounce every six hours.

Saw the animal two days later, when a marked improvement was noticed. Temperature, 102.5 F. Pulse, 60, not nearly so wiry. Respiration, 12 per minute. Appetite good. Water taken in moderate quantities. Changed the treatment by discontinuing the nitrate of potash and giving the following:

R. Sodium Salicylate, 2 oz.
Fl. Ext. Gentian, 2 oz.
Aqua, 8 oz. M.
Sig.: Give one ounce three times per day.

Did not see the mare again, but was informed that she entirely recovered in about ten days, and when due gave birth to a healthy foal.

CASE II.

Sorrel mare, eleven years old, due to foal.

*History*—Health good. Had never suffered from any disease. Had raised two colts, both of which were healthy. For several days previous to being consulted, the mare showed slight lameness, which gradually increased in severity. Loss of appetite. Seemed to drink more than usual. First noticed slight lameness in left hind leg. Was then called to prescribe for the mare.

*Surroundings*—Barn contained carriage room, one box and three single stalls. Ventilation poor, ceiling covered with frost, which was melting and dropping on the stock. Floors so wet that bedding was damp all the time.

*Feed*—Millet, hay and oat straw. The millet and oats had been seeded together, and both were headed when cut for hay. Grain ration consisted of two quarts nice clean oats as a feed three times per day.

*Symptoms*—Four or five days prior to the attack the kidneys worked freely, but at time of consultation there was but a small quantity of urine secreted. Great lameness in left hind leg. All the joints were affected, the hock, stifle and fetlock being especially sore and tender to the touch. Tucked-up appearance of the abdomen. Respiration, 12 per minute. Temperature, 102.5 degrees F. The animal would stand up part of the time, disliked to lie down, and when down would not rise unless urged to do so. The third day after the attack she foaled a healthy colt. Through carelessness of the attendant, the colt became chilled by being allowed to lie in the water which came with it, and died in thirty-six hours. The mare refused to get up, was then placed in slings, and during her endeavors to stand ruptured the tendon of the *flexor pedis perforatus* of the right hind foot from its insertion. She was then destroyed.

*Treatment*—Consisted of stimulants and salicylate of soda and nitrate of potash. There was an improvement up to the time of foaling, but at that time the symptoms became aggravated.

CASE III.

*History*—Bay mare, age unknown, had given birth to a healthy foal three weeks prior to my being called. Foal healthy at present time (seven months old).

*Surroundings*—Ventilation imperfect. Fed on millet hay and oats.

*Symptoms*—Found the animal lying down, endeavored to get her up by the use of slings, would not bear any weight on hind legs. Pain upon pressure of any of the joints of hind legs, and also in gluteal region. Temperature, 104 F. Respiration, 20. Pulse, 70. Rigors and distressed appearance.

FIG. III.

*a* Depression caused by absorption of articular cartilage.

*b* Rupture of Popliteus from its origin.

*c* Elevation and depression the result of deposition and absorption.

After thirty-six hours' treatment the attendent attempted to raise the mare and let her drop, killing her almost instantly. During the struggle she ruptured the gastrocnemious externas from its insertion.

POST-MORTEM.

I held a post-mortem on both the hind legs and found them essentially the same in morbid appearances. I will describe the lesions as they occurred in the left hind leg, those of the right leg being fully as severe The insertion of the abductor magnus was torn from its attachment and carried with it particles of bone. The portion of the bone which was removed was nearly circular in shape, a little pointed at the upper extremity. and in all covered about one and one-half square inches. The region of the popliteus was torn loose and also carried with it small particles of bone; the bone exposed was somewhat softened, it was also severed from its attachment to the capsular ligament of the stifle joint. The capsular ligament of the patella showed marked infiltrations with a diminished supply of synovial fluid; in fact, the absence of synovial fluid was well marked in all the joints, there being scarcely a trace left in some of them. The insertion of the triceps adductor femoris. the tensor fascie lata and quadriceps cruralis were also torn loose from the bone, and as was the case with the other muscles, car-

FIG IV.

*b* Absorption of cartilage

*B* Rupture of origin of Popliteus containing particles of bone.

ried with them particles of bone. The bursa at the side of the hock, through which the tendon of the peroneus muscle passed, was dry and contained scarcely a trace of synovial fluid The parts adhered to each other, showing that the condition had existed for some little time previous to death. The gastrocnemius externus was also ruptured from its attachment, allowing the hock to become flexed. The summit of the os calcis protruded through the soft tissues and skin so that it was exposed to sight, as indicated in figure. No synovial fluid found in the hock joint. The external straight ligament of the patella, the external middle inferior sesamoidean ligament, the right branch of the superior sesamoidean, the lateral ligaments of the pastern joint and the two posterior ligaments of the same joint were torn from their attachments. The capsular ligament of the fetlock joint perforated at its postero infero aspect, and also near the center of the postero internal aspect.

*The Joints*—The distal extremity of the femur presented indentations on both condyles and trochlea. On the condyles they were small, pit-like, some round, some oblong, while some were long and narrow, having the appearance of a line. The trochlea was smooth, and at its inferior portion the cartilage had become

FIG. V.

*a* Denuded bone showing result of absorption of cartilage.
*b* Ditto.

nearly worn away so that distinct grooves were noticeable where it came in contact with the patella.

*The Patella*—The texture of the patella was spongy, being not nearly so hard and compact as the average. A portion of its ligamentous attachments had given away, exposing the cancellated structure of the bone. Its posterior surface, which articulates with the trochlea of the femur, especially the innermost concavity, contained numerous indentations, exhibiting a partial destruction of the cartilage. These indentations were nearly all oval, a few of them triangular in shape, pit-like, and some of them quite deep.

In the chest, inside the lateral borders, abrupt eminences, composed wholly of cartilage, existed.

*The Tibia*—The proximal extremity showed indentations similar in character, but not nearly so numerous as those which existed on the distal extremity of the tibia, and also showed more marked deterioration than any of the other joints in the limb The cartilages in the particular grooves had completely disappeared in the center, leaving the bone exposed. The disease had not existed long enough, evidently, to allow porcelanous deposit to take place; it was somewhat roughened in character. The ridges of the astragalus contained numerous small indentations at their acute angle, and showed an almost entire absence of the articular cartilage.

*Fetlock Joint*—Changes similar to those of the femural patella and tibia joint. The pastern joint showed very little change and that on the distal extremity of the os suffraginous. All the particular surfaces of the os coronæ, of navicular and os pedis were normal with the exception of two small spots which existed on the articular surface of the os pedis where it articulates with the os corona.

## Figures and Tables

**Fig. I. f1:**
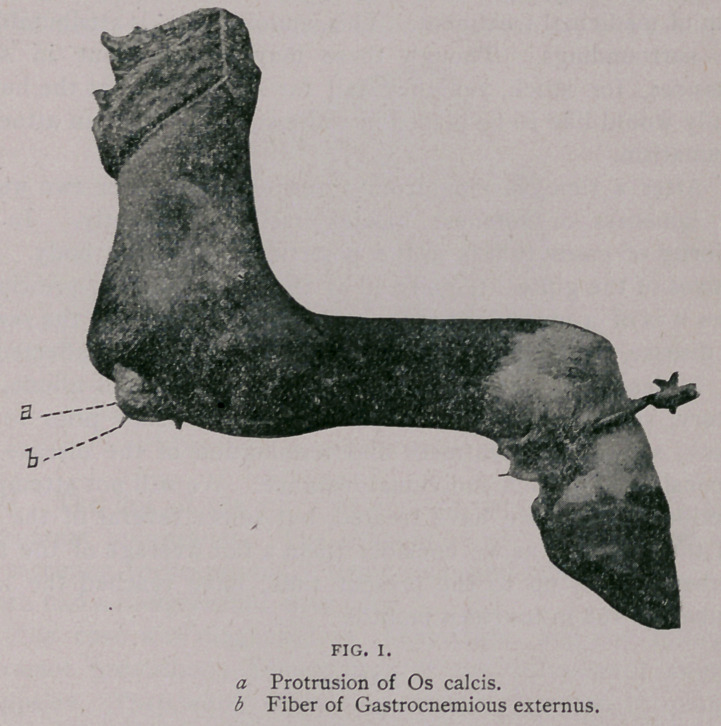


**Fig. II. f2:**
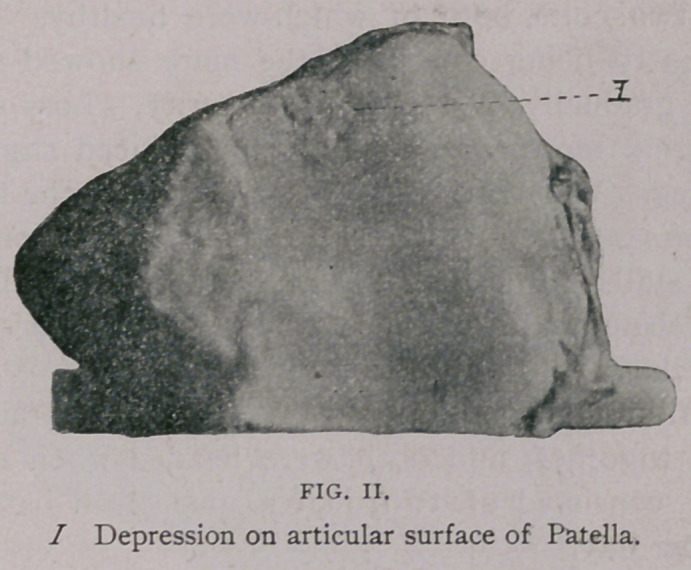


**Fig. III. f3:**
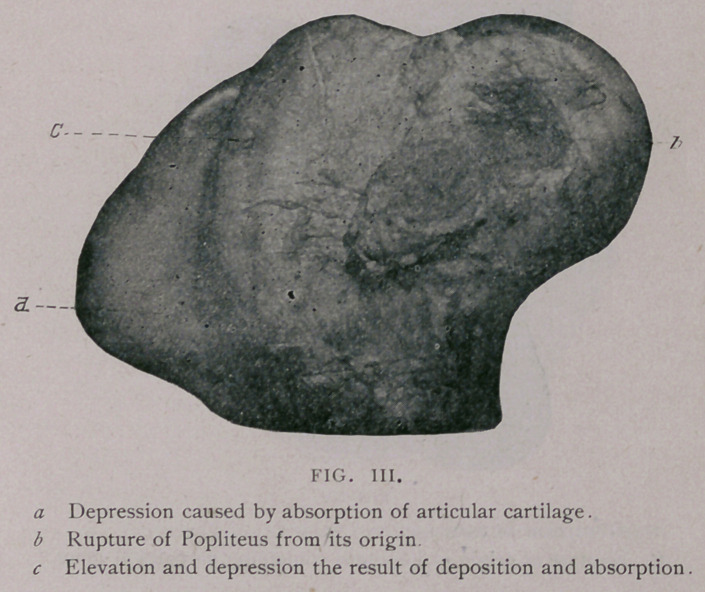


**Fig IV. f4:**
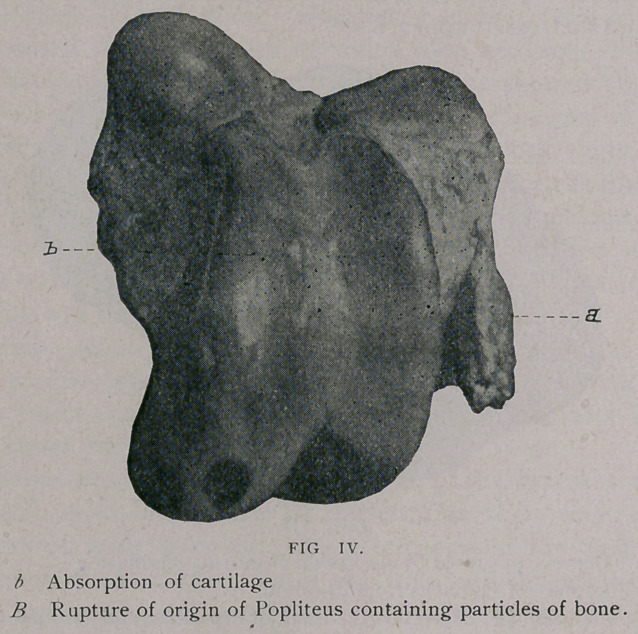


**Fig. V. f5:**